# Concordance Between [^18^F]Flortaucipir PET Visual Reads and CenTauR-Based Quantification

**DOI:** 10.2967/jnumed.126.272722

**Published:** 2026-11

**Authors:** Stamatia Karagianni, Alexis Moscoso, Sheelakumari Raghavan, Martijn van Essen, Ismini Mainta, Valle Camacho, Omar Rodríguez-Fonseca, Jesús Silva-Rodríguez, Andrés Perissinotti, Yuna Gu, Jihwan Yun, Nicolai Franzmeier, Michel J. Grothe, Sang Won Seo, Giovanni B. Frisoni, Valentina Garibotto, Christopher G. Schwarz, Ronald C. Petersen, Clifford R. Jack, Prashanthi Vemuri, Michael Schöll

**Affiliations:** 1 Wallenberg Centre for Molecular and Translational Medicine, University of Gothenburg, Gothenburg, Sweden;; 2 Department of Psychiatry and Neurochemistry, Institute of Neuroscience and Physiology, Sahlgrenska Academy, University of Gothenburg, Gothenburg, Sweden;; 3 Nuclear Medicine Department and Molecular Imaging Group, Instituto de Investigación Sanitaria de Santiago de Compostela, Santiago de Compostela, Spain;; 4 Department of Radiology, Mayo Clinic, Rochester, Minnesota;; 5 Department of Clinical Physiology/Nuclear Medicine, Sahlgrenska University Hospital, Gothenburg, Sweden;; 6 Department of Molecular and Clinical Medicine, Institute of Medicine, Sahlgrenska Academy, Gothenburg University, Gothenburg, Sweden;; 7 Division of Nuclear Medicine, Geneva University Hospitals, Geneva, Switzerland;; 8 Department of Nuclear Medicine, Hospital de la Santa Creu i Sant Pau, Universitat Autònoma de Barcelona, Barcelona, Spain;; 9 Nuclear Medicine Department, Lucus Augusti University Hospital, Lugo, Spain;; 10 Reina Sofía Foundation Alzheimer’s Centre, CIEN Foundation, ISCIII, Madrid, Spain;; 11 Nuclear Medicine Department, Hospital Clínic Barcelona–IDIBAPS, Barcelona, Spain;; 12 Biomedical Research Networking Centre of Bioengineering, Biomaterials and Nanomedicine, ISCIII, Barcelona, Spain;; 13 Samsung Alzheimer Research Center, Research Institute for Future Medicine, Samsung Medical Center, Seoul, Korea;; 14 Department of Neurology, Kyung Hee University Hospital, Kyung Hee University College of Medicine, Seoul, Korea;; 15 Institute for Stroke and Dementia Research, LMU University Hospital, LMU Munich, Munich, Germany;; 16 Munich Cluster for Systems Neurology, Munich, Germany;; 17 Department of Clinical Research Design and Evaluation, Samsung Advanced Institute for Health Science and Technology, Sungkyunkwan University, Seoul, Korea;; 18 Department of Health Science and Technology, Samsung Advanced Institute for Health Science and Technology, Sungkyunkwan University, Seoul, Korea;; 19 Geneva Memory Center, Department of Rehabilitation and Geriatrics, Geneva University Hospitals, Geneva, Switzerland;; 20 Laboratory of Neuroimaging of Aging, University of Geneva, Geneva, Switzerland;; 21 Faculty of Medicine, University of Geneva and Center for Biomedical Imaging, Switzerland;; 22 Department of Neurology, Mayo Clinic, Rochester, Minnesota; and; 23 Department of Neuropsychiatry, Sahlgrenska University Hospital, Mölndal, Sweden

**Keywords:** tau PET imaging, [^18^F]flortaucipir, visual interpretation, CenTauR quantification, Alzheimer disease

## Abstract

Tau PET imaging with [^18^F]flortaucipir allows for the visualization and mapping of aggregated tau deposits, a key neuropathologic feature of Alzheimer disease (AD). A visual interpretation method for [^18^F]flortaucipir was approved by the Food and Drug Administration and European Medicines Agency and has been implemented for a standardized, clinically usable definition of tau PET positivity. The CenTauR_z_ method, on the other hand, offers the possibility to harmonize the definition of tau PET positivity across different radiotracers using quantitative metrics, but its concordance with clinically relevant [^18^F]flortaucipir visual reads remains unclear. **Methods:** A convenience sample of 3991 participants, including cognitively unimpaired (CU) and cognitively impaired (CI) individuals (i.e., those with mild cognitive impairment or AD dementia), underwent [^18^F]flortaucipir PET imaging. Each [^18^F]flortaucipir scan was assessed by 3 trained readers using the approved visual interpretation method and quantified using the CenTauR_z_ quantification pipeline in different regions of interest (ROIs). Concordance between positive visual reads and CenTauR_z_-defined positivity (i.e., >2 on the CenTauR_z_ scale) was assessed using Cohen κ. Receiver-operating-characteristic (ROC) analysis evaluated the discriminative power of continuous CenTauR_z_ values in distinguishing between negative and positive visual reads. Generalized additive models examined clinical progression on the basis of visual and CenTauR_z_-based assessments of tau PET positivity. **Results:** Concordance between visual reads and CenTauR_z_-based assessments of tau PET positivity was moderate, particularly in CU individuals (κ = 0.23–0.55 for CU participants, κ = 0.57–0.82 for CI participants, depending on the ROI). ROC analysis revealed that the agreement remained moderate, independent of the CenTauR_z_ cut point used (area under the ROC curve, 0.72–0.87 vs. 0.87–0.97 for CU and CI individuals, respectively). Among discordant cases, participants with visually positive/CenTauR_z_-negative tau PET scans were more frequently amyloid-positive and exhibited faster clinical progression compared with visually negative/CenTauR_z_-positive individuals. **Conclusion:** Our findings highlight the relatively limited agreement between the visual assessment of [^18^F]flortaucipir PET images and CenTauR_z_-based quantification, particularly in CU individuals. Participants with visually positive/CenTauR_z_-negative tau PET scans showed a high frequency of amyloid positivity and faster clinical progression, suggesting that visual reads are more sensitive to heterogeneous, clinically relevant tau accumulation patterns not captured by ROI-based methods. These findings underscore the need for new quantification approaches to better capture the complex patterns of tau deposition, important for early AD detection and monitoring.

## Visual Abstract

**Figure:**
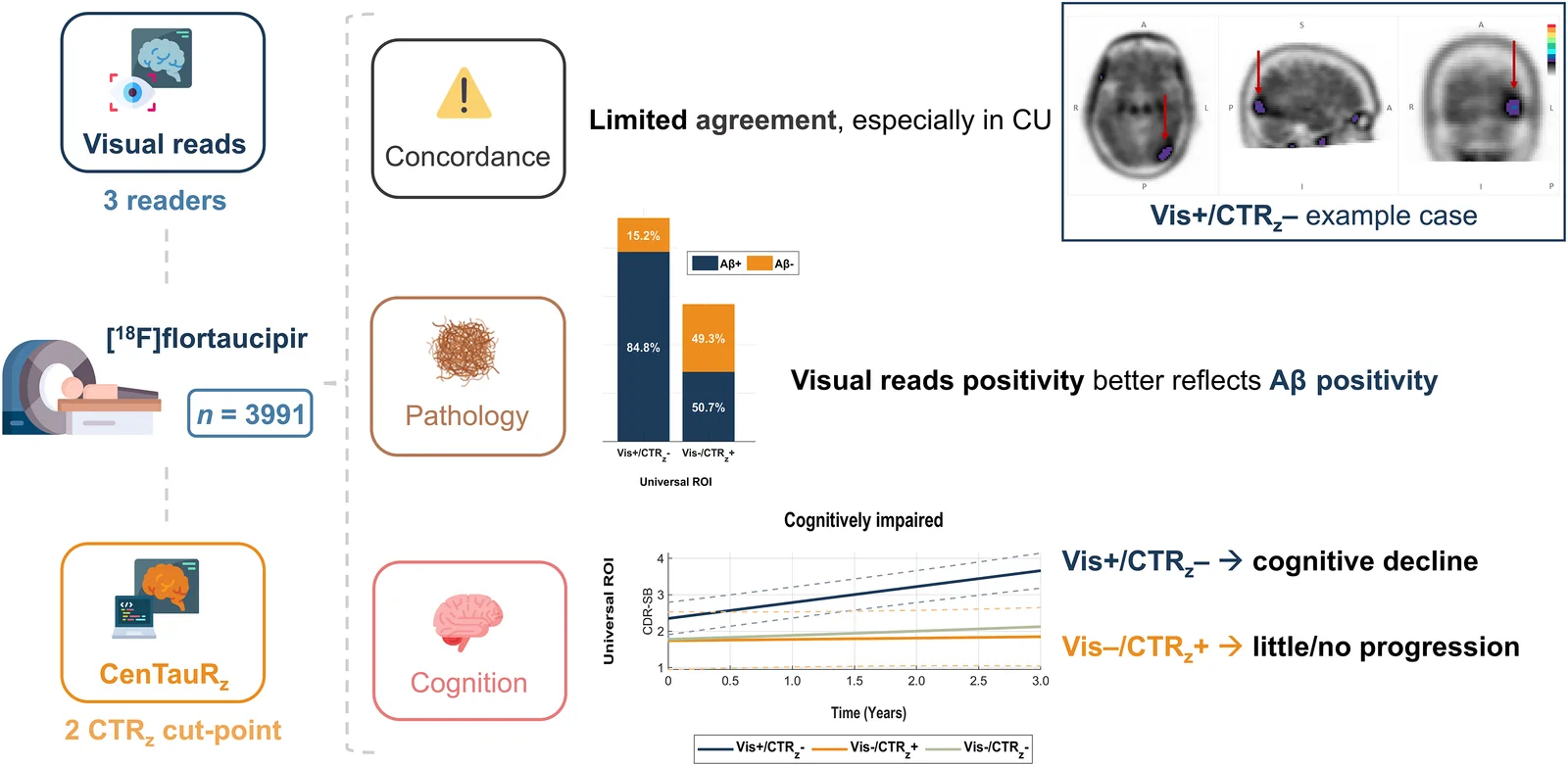

The advent of tau PET imaging has enabled the in vivo visualization, mapping, and quantification of aggregated tau deposits (1,2), a key neuropathologic hallmark of Alzheimer disease (AD) closely linked to neurodegeneration and clinical symptoms (3). Recently, tau PET imaging has transitioned into clinical practice with the approval of the tau PET tracer [^18^F]flortaucipir by both the Food and Drug Administration (FDA) (4) and the European Medicines Agency (EMA) (5), marking a milestone in AD diagnostics. For clinical application, the FDA/EMA labeling for [^18^F]flortaucipir requires a standardized visual interpretation method for establishing tau PET positivity (1,4,5). With this method, a visually positive [^18^F]flortaucipir PET scan reflects advanced neurofibrillary tangles (Braak stages V and VI, which corresponds to B3 in the National Institute on Aging–Alzheimer’s Association guidelines) (1), closely associated with AD-specific symptoms and short-term clinical decline (6–9). Notably, visual interpretation remains the only standardized, clinically applicable definition of tau PET positivity with an established neuropathologic correlate—Braak stages V and VI.

Currently, [^18^F]flortaucipir is the only tau PET tracer approved for clinical use (4,5), although several other tau PET tracers are available in research settings and likely to enter clinical practice in the near future (10,11). This evolving tracer landscape underscores the need for harmonized quantitative approaches that enable tracers’ comparability. Efforts toward establishing a universal definition of tau PET positivity have primarily focused on harmonizing quantitative metrics across the different tau PET radiotracers. Currently, a tau PET harmonization initiative, the CenTauR project (https://www.gaain.org/centaur-project), has recently been launched to address this need (12,13). Conceptually analogous to the Centiloid approach for amyloid-β peptide (Aβ) PET (14), CenTauR relies on a dedicated tau PET quantification pipeline to estimate SUV ratios (SUVRs) (12). These values are subsequently transformed into a unified scale—CenTauR_z_ (12), or the currently developed CenTauR scale (13)—using linear equations. A tracer-independent definition of tau PET positivity could then be derived by applying a specific CenTauR_z_ or CenTauR cutoff value.

Although visual reads and the CenTauR scale aim to classify tau PET scans as positive or negative to determine the predefined tau burden, their comparability may be limited by significant differences in how they define tau positivity. A key methodologic difference lies in the fact that FDA/EMA-approved visual reads for [^18^F]flortaucipir do not consider a signal in the anterior and mesial temporal cortex as positive (1) (Fig. 1B). In contrast, the most widely used quantification methods, including the CenTauR quantification pipeline, rely heavily on considering anterior temporal cortex regions of interest (ROIs) (15,16) (Fig. 1A). Another important distinction is that visual reads allow for the assessment of brain regions beyond the temporal cortex, enabling detection of an isolated signal, such as in the occipital or parietal region that would not be captured by commonly used predefined temporal ROIs. These considerations suggest that tau PET positivity should not be presumed to be interchangeable between these methods.

**FIGURE 1.**
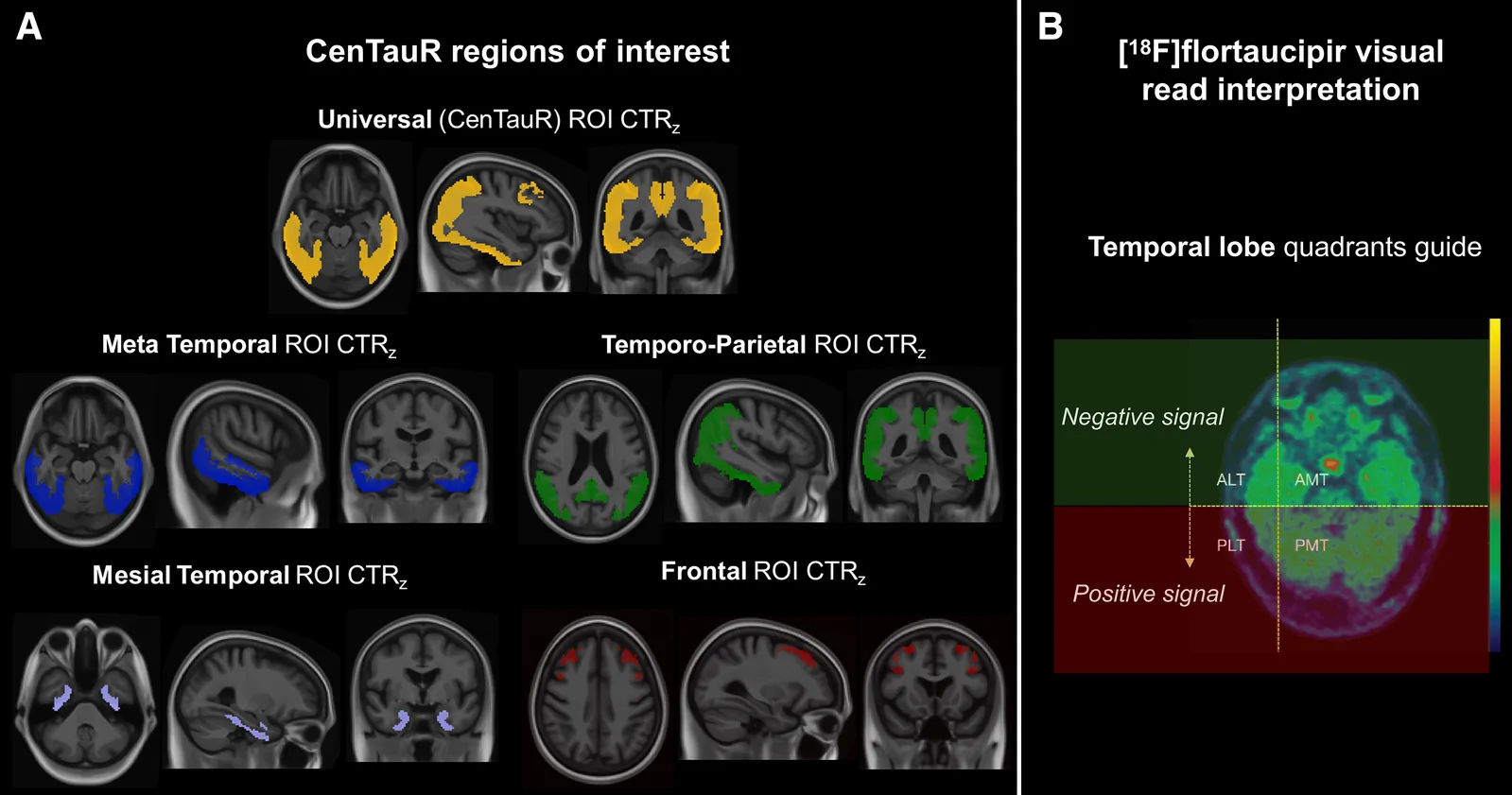
CenTauR ROIs and temporal lobe quadrants for visual interpretation of [^18^F]flortaucipir. Panel A shows 5 different ROIs defined by CenTauR project (https://www.gaain.org/centaur-project). Panel B shows adapted version of temporal lobe quadrants guide using on FDA/EMA-approved visual interpretation method for [^18^F]flortaucipir (4,5). Elevated signal in anterolateral temporal (ALT) and anterior mesial temporal (AMT) quadrants is interpreted as negative, whereas elevated signal in posterolateral temporal (PLT) and posterior mesial temporal (PMT) quadrants is interpreted as positive.

To date, studies comparing FDA/EMA-approved [^18^F]flortaucipir PET visual reads with quantification methods have been limited by relatively small sample sizes or using post hoc–derived positivity cutoffs (7), which may have yielded overoptimistic concordance estimates. This study aims to address these limitations by systematically examining the concordance between the clinically approved visual read method for [^18^F]flortaucipir PET and CenTauR_z_-based quantification in a large sample of individuals across the clinical AD spectrum. Additionally, we explored the biologic and clinical correlations of each definition of tau PET positivity and investigated the implications of discordance between the methods.

## MATERIALS AND METHODS

### Participants

We included 3991 participants from 10 different cohorts (Mayo Clinic Study of Aging, Alzheimer’s Disease Neuroimaging Initiative, Anti-Amyloid Treatment in Asymptomatic Alzheimer’s Study, OASIS-3 trial, Avid Radiopharmaceuticals ^18^F-AV-1451-A05 study, National Alzheimer’s Coordinating Center’s Standardized Centralized Alzheimer’s and Related Dementias Neuroimaging initiative, Mayo Clinic Alzheimer’s Disease Research Center, Geneva Memory Center, Harvard Aging Brain Study, and Samsung Medical Center). Inclusion criteria and imaging acquisition protocols can be found in the Supplemental Methods, available at http://jnm.snmjournals.org. In brief, participants were eligible for inclusion if they had undergone [^18^F]flortaucipir PET and T1-weighted MRI. Additionally, included participants were required to be either cognitively unimpaired (CU) or have a clinical diagnosis of mild cognitive impairment (17) or AD dementia (18), as determined by established clinical criteria within each cohort. Most participants (*n* = 3842, 96.3%) had also undergone Aβ PET imaging with [^11^C]PiB, [^18^F]florbetapir, or [^18^F]florbetaben, and Aβ PET quantification results were expressed in Centiloids (14). Aβ PET positivity was defined by applying the neuropathologically validated cut point of 24.4 Centiloids (19). Follow-up clinical assessments were available for 3040 participants, with a median follow-up time of 3.23 ± 1.89 y (maximum, 8.25 y). Study participants’ characteristics are summarized in Table 1.

**TABLE 1.** Characteristics of Study Participants

Baseline characteristic	Cognitively unimpaired	Mild cognitive impairment	AD dementia
*n*	2622	863	506
Age (y)	69.5 (21–95)	73 (43.9–97)	71.6 (28–95)
Female	1415 (54)	374 (43.3)	275 (54.3)
Aβ PET			
Aβ positive (>24.4 Centiloids) (19)	932 (37.1)	466 (55.8)	437 (88.6)
Not assessed	108	28	13
Positive tau PET visual read	262 (10)	363 (42.1)	418 (82.6)
Tau PET CenTauR_z_ quantification			
Universal ROI positive*	249 (9.5)	318 (36.8)	389 (76.9)
Meta Temporal ROI positive*	221 (8.4)	326 (37.8)	393 (77.7)
Mesial Temporal ROI positive*	326 (12.4)	379 (43.9)	418 (82.6)
Temporo-Parietal ROI positive*	235 (9)	312 (36.2)	388 (76.7)
Frontal ROI positive*	185 (7.1)	211 (24.4)	325 (64.2)
Participants with available CDR-SB follow-up data	2149 (82)	581 (67.3)	310 (61.3)
CDR-SB follow-up time (y)^†^	4	1.6	1.5

* Positivity was defined as CenTauR_z_ score of greater than 2.

† Expressed as the median.

Qualitative data expressed as number, followed by percentage in parentheses; continuous data expressed as mean, followed by range in parentheses.

The use of all data for the current study was approved by the local institutional review boards for human research at each participating institution. All participants provided written informed consent.

### [^18^F]Flortaucipir PET Visual Reads

[^18^F]flortaucipir PET scans were classified as either positive or negative using the FDA/EMA-approved visual interpretation method (4). According to this, a scan is considered positive if there is increased signal in the posterolateral temporal, occipital, or parietal/precuneus cortex, with or without elevated signal in frontal areas. Of note, elevated signal in the anterior and mesial temporal cortex does not contribute to a positive read (Fig. 1B; Supplemental Methods).

Visual interpretation of [^18^F]flortaucipir PET images was performed by a team of 12 newly trained readers (20). In total, 3991 tau PET scans were processed for visualization using an in-house developed pipeline and visually assessed by 1 reader, for a minority of scans, because of limited reader availability (*n* = 402, 10.1%) or by 3 readers, for the majority (*n* = 3589, 90%), masked to any clinical and biomarker information (Supplemental Methods). The robustness of the visual interpretation procedure has been previously validated (20) and demonstrated excellent interrater agreement in the present study (mean Cohen κ = 0.92; range, 0.90–0.94).

### [^18^F]Flortaucipir PET Quantification

[^18^F]flortaucipir PET scans were quantified using the SPM8-based CenTauR_z_ “standard” quantification pipeline (https://www.gaain.org/centaur-project; Supplemental Methods). The pipeline provides an inferior cerebellar cortex ROI as reference region, as well as 5 target cortical ROIs (Universal, Meta Temporal, Mesial Temporal, Temporo-Parietal, and Frontal), all in Montreal Neurologic Institute space (Fig. 1A). SUVRs are converted into CenTauR_z_ values, defined as SUVR *z*-scores using a specific sample of Aβ-negative CU individuals as the reference population (12).

A cut point of 2 on CenTauR_z_ has been proposed to define tau PET positivity within each predefined ROI (12), corresponding to 2 SD above the mean of a sample of Aβ-negative CU individuals, a criterion commonly used in research studies (16).

### Clinical Assessments

Clinical progression was assessed using the Clinical Dementia Rating Sum of Boxes (CDR-SB) score (21), obtained through interviews with patients and their care partners, capturing both cognition and function. Scores ranged from 0 to 18, with higher scores indicating increased dementia severity.

### Statistical Analysis

Agreement between CenTauR_z_-based tau positivity (CTR_z_+) and visual read–assessed tau positivity (Vis+) and across the various ROIs was evaluated using Cohen κ. To further assess concordance without relying on the prespecified 2 CenTauR_z_ cut point, we used the area under the receiver-operating-characteristic curve (AUC) to quantify the ability of continuous CenTauR_z_ values to distinguish between positive and negative visual reads. To further explore the impact of cut point selection, we computed optimal cut points for discrimination by maximizing the Youden index and recomputed the corresponding κ coefficients.

To characterize discordance patterns, we quantified the frequency of Vis+/CenTauR_z_-based tau negativity (CTR_z_−) and visual read–assessed tau negative (Vis−)/CTR_z_+ cases across ROIs and compared their corresponding Aβ positivity rates. Group differences in Aβ positivity were assessed using χ^2^ tests. To explore the clinical implications of discordance, we modeled CDR-SB trajectories over time using generalized additive models. The models included a time cubic spline term with 3 knots for each Vis/CTR_z_ group and subject-specific random intercepts. Discordant groups (Vis+/CTR_z_−, Vis−/CTR_z_+) were compared with the concordant negative group (Vis−/CTR_z_−). Group differences were summarized as estimated differences in CDR-SB (ΔCDR-SB) at 1.5 y in CI individuals and 5 y in CU individuals, reflecting typical durations of symptomatic and asymptomatic AD clinical trials, respectively. All analyses were performed separately for CU and CI (groups with mild cognitive impairment and AD dementia combined) individuals.

## RESULTS

### Concordance Between Visual Reads and CenTauR_z_ Quantification Outcome

Figure 2 shows the concordance results between [^18^F]flortaucipir PET Vis+ and CTR_z_+ (CTR_z_ > 2). Agreement was modest for CU individuals (κ = 0.23–0.55, depending on the ROI) but notably higher for CI participants (κ = 0.57–0.82). Cohort-level analyses showed consistent patterns across datasets, although effect sizes varied (Supplemental Table 1). Although performance was relatively similar across the investigated ROIs, the Frontal ROI stood out by yielding markedly lower concordance (κ = 0.23 and κ = 0.57 for CU and CI individuals, respectively). These results were similar when the analyses were restricted to Aβ-positive individuals (κ = 0.22–0.58 for Aβ-positive CU individuals and κ = 0.38–0.71 for Aβ-positive CI individuals; Fig. 3).

**FIGURE 2.**
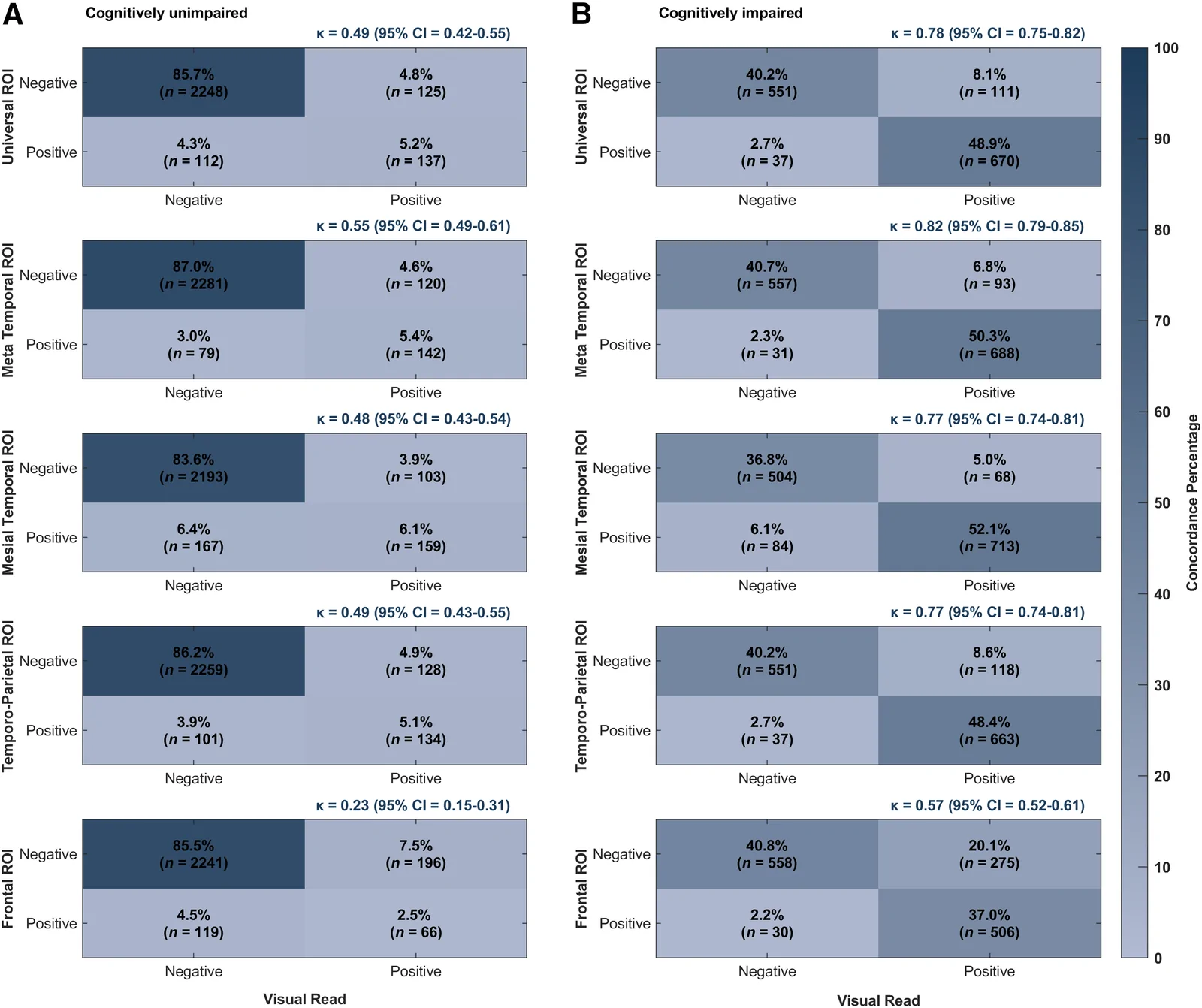
Concordance between visual reads for [^18^F]flortaucipir and CenTauR_z_-based quantification. Shown are contingency matrices for agreement between visual read–based and CenTauR_z_-based definitions of tau PET positivity for [^18^F]flortaucipir. CTR_z_+ was defined using prespecified cut point (>2 CTR_z_) and investigated in 5 different regions of interest provided by CenTauR project (https://www.gaain.org/centaur-project).

**FIGURE 3.**
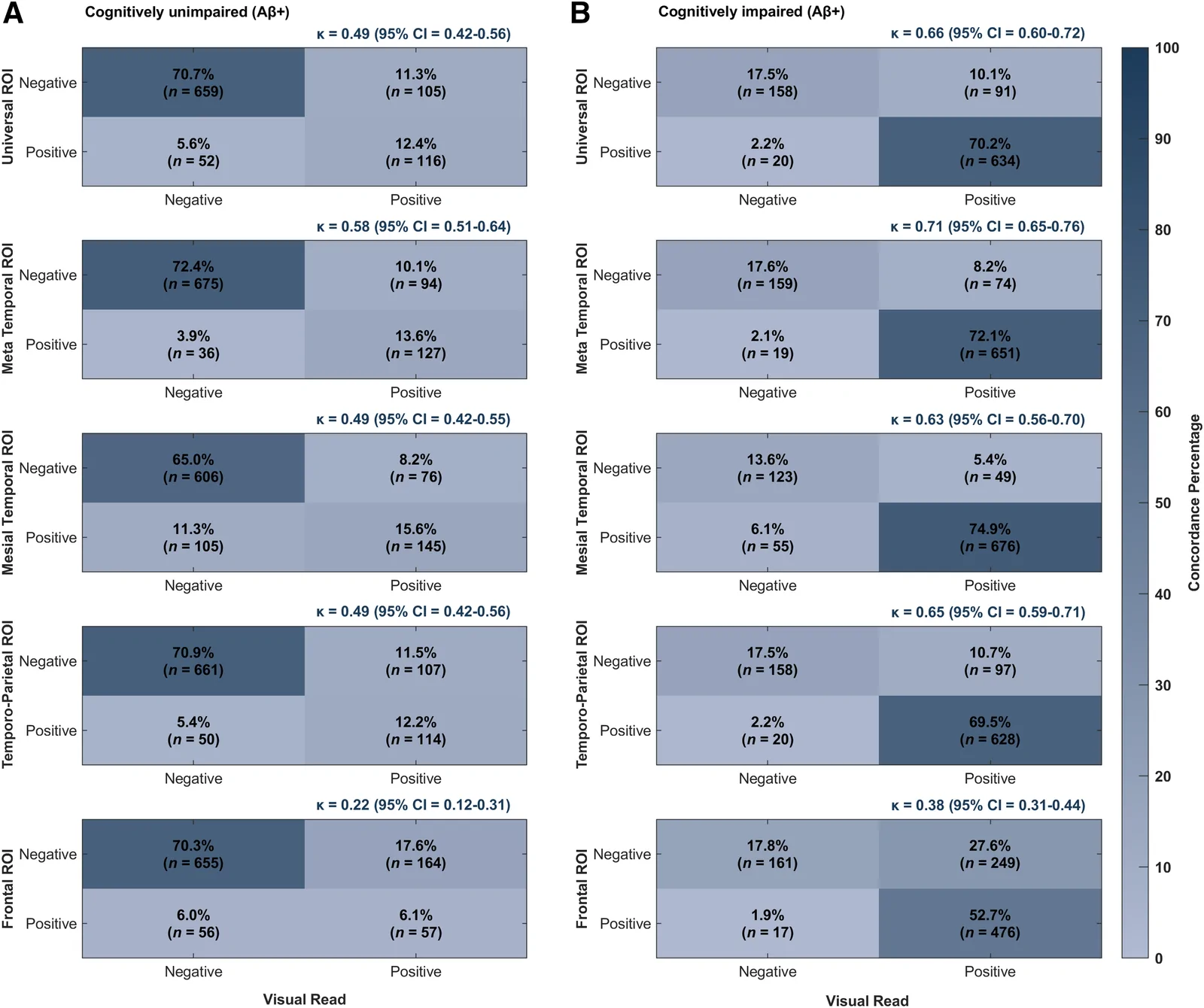
Concordance between visual reads for [^18^F]flortaucipir and CenTauR_z_-based quantification among Aβ-positive individuals. Shown are contingency matrices for agreement between visual read– and CenTauR_z_-based definitions of tau PET positivity for [^18^F]flortaucipir among Aβ-positive individuals. CTR_z_+ was defined using prespecified cut point (>2 CTR_z_) and investigated in 5 different regions of interest provided by CenTauR project (https://www.gaain.org/centaur-project).

Except for the Mesial Temporal ROI, the primary source of discordance between the methods was the high proportion of Vis+ not classified as positive by CenTauR_z_ quantification, ranging from 39% (103/262) to 75% (196/262) of the Vis+ CU participants and from 9% (68/781) to 35% (275/781) for Vis+ CI individuals (Fig. 2). Qualitatively, we observed that most Vis+/CTR_z_− cases displayed focally and asymmetrically elevated signal, typically in temporoparietal (Fig. 4A) or occipital (Fig. 4B) regions.

**FIGURE 4.**
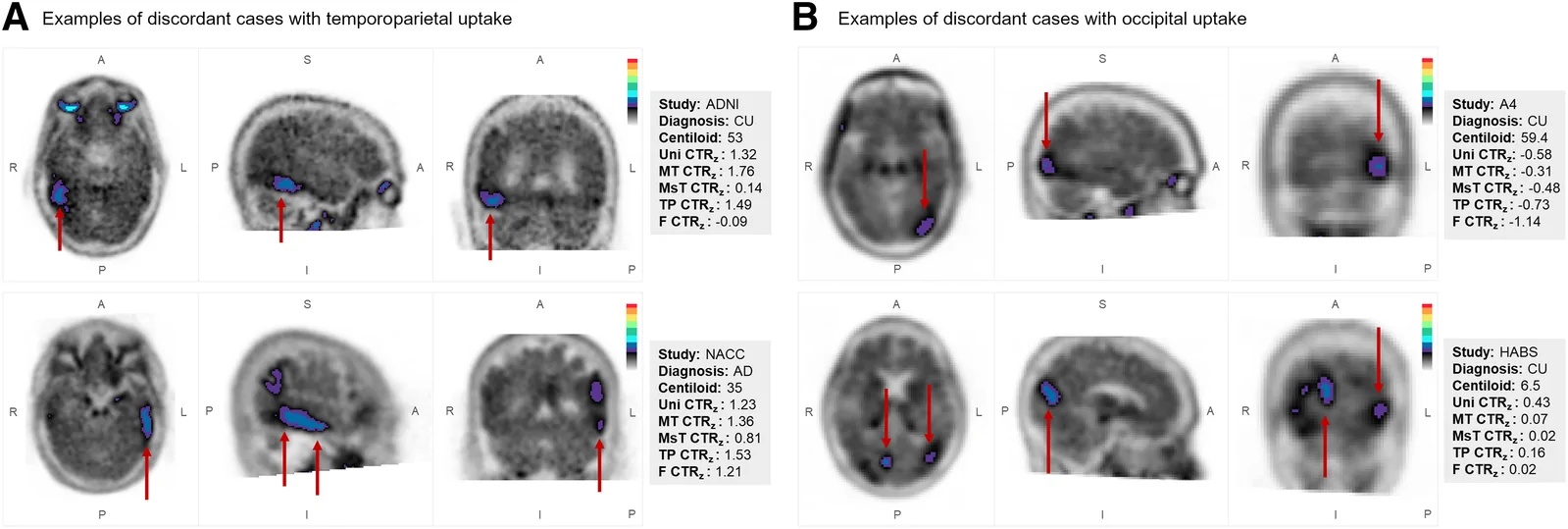
Examples of study participants with Vis+/CTR_z_− results on [^18^F]flortaucipir PET. Per FDA/EMA-approved visual interpretation method for [^18^F]flortaucipir PET, colors above gray scale portion of scale represent elevated [^18^F]flortaucipir uptake (red arrows). Panel A shows examples of 2 study participants from Alzheimer’s Disease Neuroimaging Initiative (ADNI) and National Alzheimer’s Coordinating Center (NACC) with elevated focal temporal or temporoparietal signal. Panel B shows examples of 2 study participants from Anti-Amyloid Treatment in Asymptomatic Alzheimer’s (A4) study, and Harvard Aging Brain Study (HABS) with isolated elevated occipital signal. All participants in panels A and B were CTR_z_− (≤ 2) on 5 CenTauR ROIs. F = Frontal ROI; MsT = Mesial Temporal ROI; MT = Meta Temporal ROI; TP = Temporo-Parietal ROI; Uni = Universal ROI.

Receiver-operating-characteristic (ROC) curve analysis examining the accuracy of continuous CenTauR_z_ values in distinguishing between Vis− and Vis+ reads also revealed moderate to good concordance, independent of the cut point used (AUC, 0.72–0.87 for CU and 0.87–0.97 for CI participants; Table 2). When using Youden-based cut points from the previous ROC analysis, κ coefficients remained similarly limited (Supplemental Table 2). Of note, in the CI group, optimal cut points for the Universal, Meta Temporal, and Temporo-Parietal ROIs were highly similar to the previously proposed CenTauR_z_ cut point of 2 (2.06, 1.92, 1.91 for Universal, Meta Temporal, and Temporo-Parietal, respectively). Mild cognitive impairment– and AD dementia–stratified analyses showed similar results across ROIs (Supplemental Table 3), suggesting no major influence of AD ceiling effects.

**TABLE 2.** AUCs for Discriminating Between Negative and Positive [^18^F]Flortaucipir Visual Reads for Each CenTauR ROI

	AUC (95% CI)				
Clinical diagnosis	Universal ROI	Meta Temporal ROI	Mesial Temporal ROI	Temporo-Parietal ROI	Frontal ROI
Cognitively unimpaired	0.85 (0.82–0.88)	0.87 (0.84–0.90)	0.86 (0.83–0.89)	0.84 (0.81–0.87)	0.72 (0.69–0.75)
Cognitively impaired*	0.95 (0.94–0.96)	0.97 (0.96–0.97)	0.95 (0.94–0.96)	0.95 (0.94–0.96)	0.87 (0.85–0.89)
Cognitively unimpaired (Aβ positive)	0.83 (0.79–0.86)	0.85 (0.82–0.88)	0.83 (0.80–0.86)	0.82 (0.78–0.85)	0.68 (0.64–0.72)
Cognitively impaired* (Aβ positive)	0.94 (0.92–0.96)	0.95 (0.94–0.97)	0.92 (0.90–0.94)	0.94 (0.92–0.95)	0.85 (0.82–0.87)

* Both participants with mild cognitive impairment and Alzheimer disease dementia were included.

Quantities in brackets parentheses are bootstrap-derived 95% CIs.

### Visual Reads vs. CenTauR_z_-Based Quantification for Detecting Aβ-Related Tau PET Positivity

Figure 5 displays the proportion of tau PET–positive individuals as defined using visual reads or CenTauR_z_ quantification, stratified by Aβ status. Visual reads yielded the lowest positivity rate among Aβ-negative CU participants (2.1%; 95% CI, 1.4%–2.8%), but a high positivity rate in Aβ-positive CU individuals (23.7%; 95% CI, 21%–26.4%), which was surpassed only by the Mesial Temporal ROI in CenTauR_z_ (26.8%; 95% CI, 24%–29.6%). A similar pattern was observed in CI individuals, with visual reads yielding a low positivity rate in Aβ-negative CI participants (8%; 95% CI, 5.4%–10.6%), comparable to some CenTauR ROIs, but higher positivity rates in Aβ-positive CI participants (80.3%; 95% CI, 77.7%–82.9%).

**FIGURE 5.**
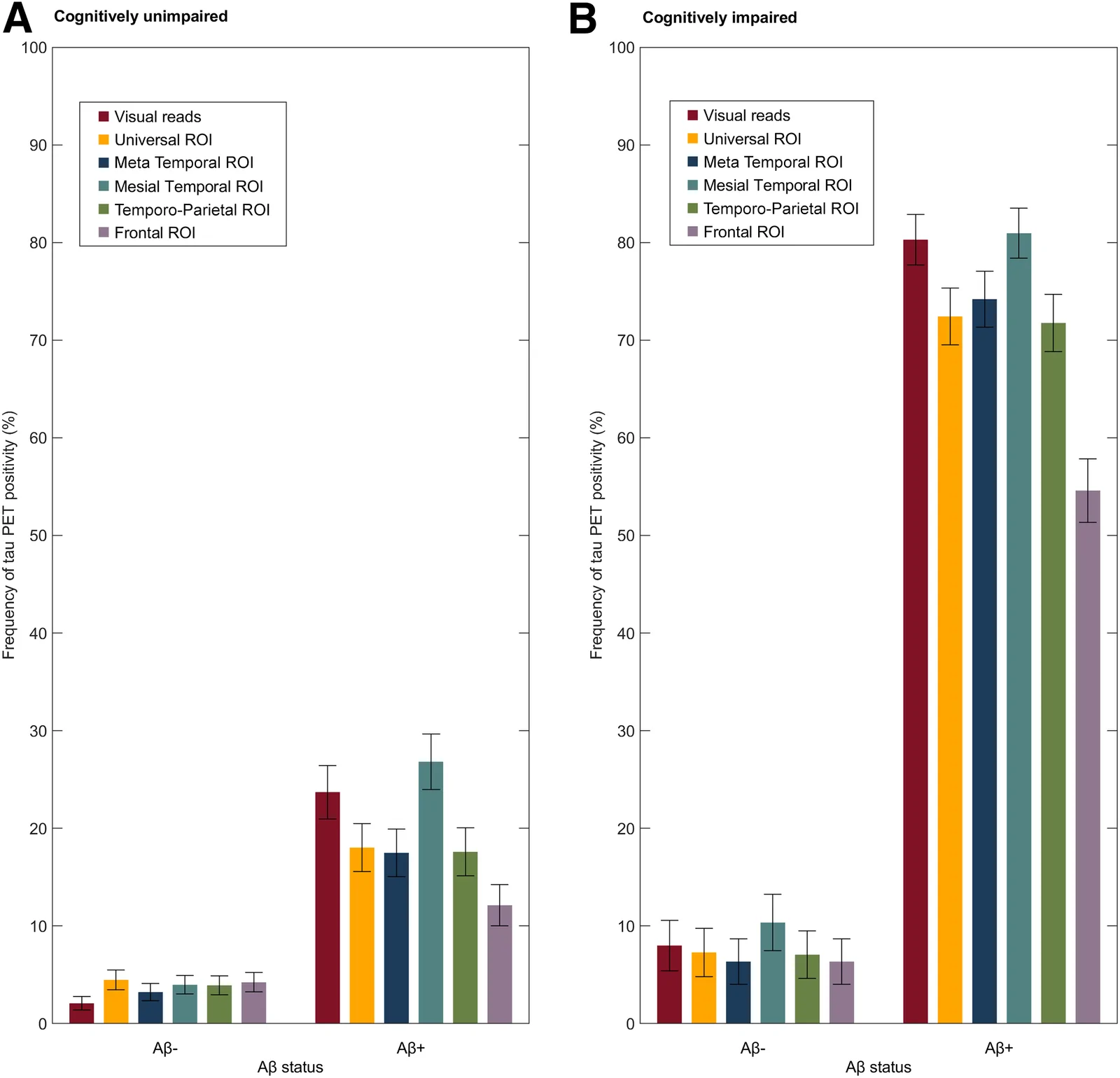
Positivity rates for visual read–based and CenTauR_z_-based definitions of tau PET positivity across Aβ-negative and Aβ-positive individuals. Proportion of tau PET–positive CU (A) and CI (B) individuals for each positivity definition are shown.

### Clinical Implication of Discordance Between Vis+ and CTR_z_+

Figure 6 shows the number of cases with discordant Vis/CTR_z_ tau PET positivity statuses. Except for the Mesial Temporal ROI, Vis+/CTR_z_− cases were approximately twice as frequently observed as those with a Vis−/CTR_z_+ profile. Moreover, a high proportion of the Vis+/CTR_z_− individuals were Aβ positive (75.8%–90.2%), whereas those with a Vis−/CTR_z_+ profile had significantly lower rates of Aβ positivity (50.3%–66.7%, *P* < 0.001). Notably, Aβ PET positivity rate in the Vis−/CTR_z_+ group was similar to the overall Aβ PET positivity observed in the full sample (47.8%, 1835/3842).

**FIGURE 6.**
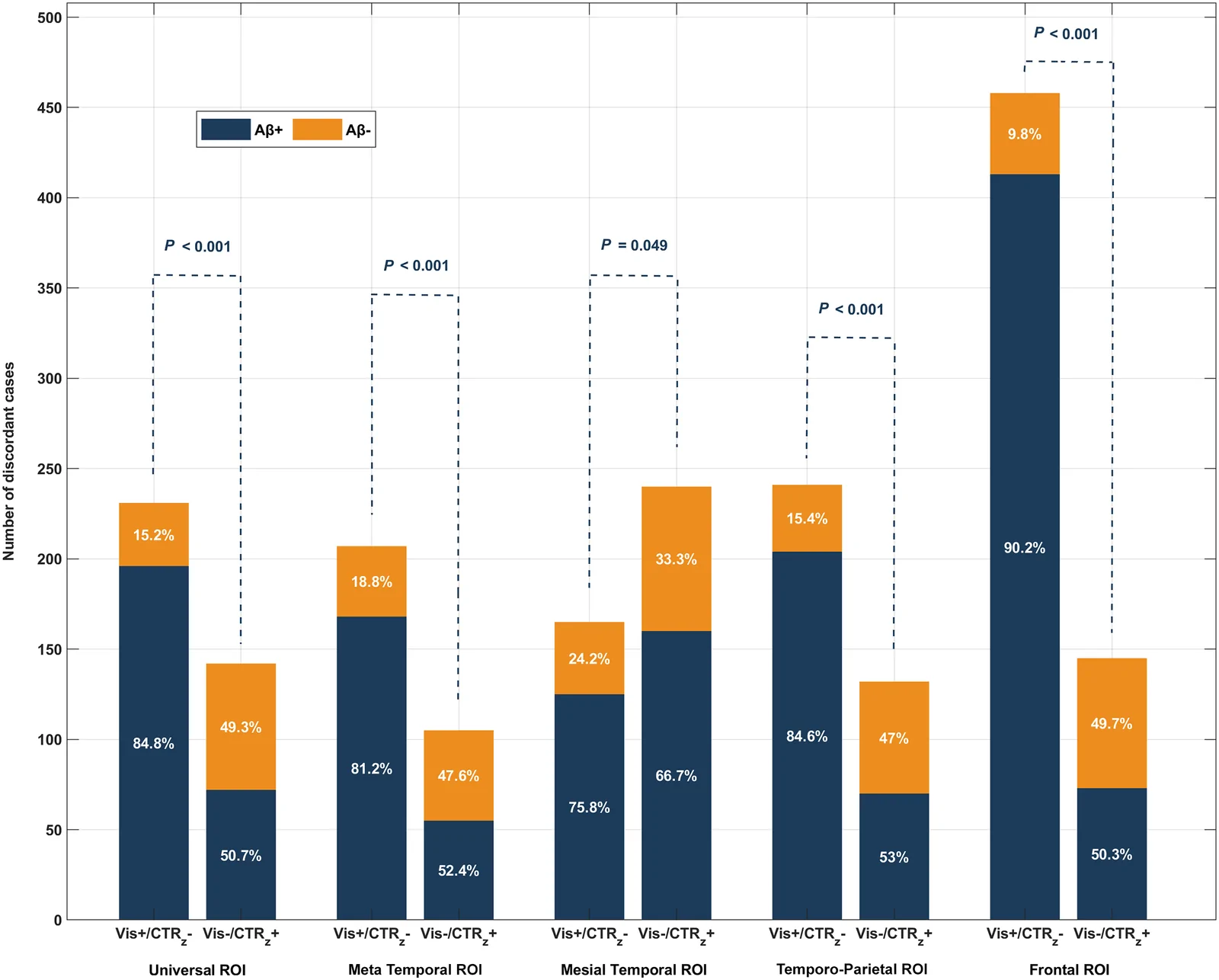
Number of cases with discordant results (Vis+/CTR_z_− and Vis−/CTR_z_+ cases) between visual read–based and CenTauR_z_-based definitions of tau PET positivity. Percentages within bars indicate proportion of Aβ-positive and Aβ-negative individuals. Reported *P* values are from χ^2^ tests comparing proportion of Aβ-positive cases between Vis+/CTR_z_− group and corresponding Vis−/CTR_z_+ group.

Discordance also entailed differential clinical trajectories. Vis+/CTR_z_−individuals exhibited significantly greater clinical decline at follow-up than the corresponding Vis−/CTR_z_− reference group for all ROIs and diagnostic groups (ΔCDR-SB for CU at 5-y follow-up, 0.46–1.11; ΔCDR-SB for CI participants at 1.5-y follow-up, 1.06–1.65; Fig. 7; Supplemental Table 4). Similar findings were observed for CU participants with a Vis−/CTR_z_+ profile, although this discordance profile showed smaller differences with the Vis−/CTR_z_− reference group than those observed for the Vis+/CTR_z_− group (ΔCDR-SB at 5-y follow-up: 0.28–0.60; Fig. 7A; Supplemental Table 4). Moreover, there were only small differences in the CDR-SB trajectories between CI participants with a Vis−/CTR_z_+ profile and the trajectories of the Vis−/CTR_z_− group for most of the studied ROIs (ΔCDR-SB at 1.5-y follow-up, −0.33 to 0.88; Fig. 7B; Supplemental Table 4), with some estimates showing small negative values within confidence intervals spanning zero. Generally, individuals with a Vis+/CTR_z_− profile had worse clinical outcomes than those with a Vis−/CTR_z_+ profile (Meta Temporal: ΔCDR-SB for the CU group at 5-y follow-up, 0.26; ΔCDR-SB for the CI group at 1.5-y follow-up, 1.14; Universal: ΔCDR-SB for the CU group at 5-y follow-up, 0.57; ΔCDR-SB for the CI group at 1.5-y follow-up, 1.21) (Supplemental Table 4).

**FIGURE 7.**
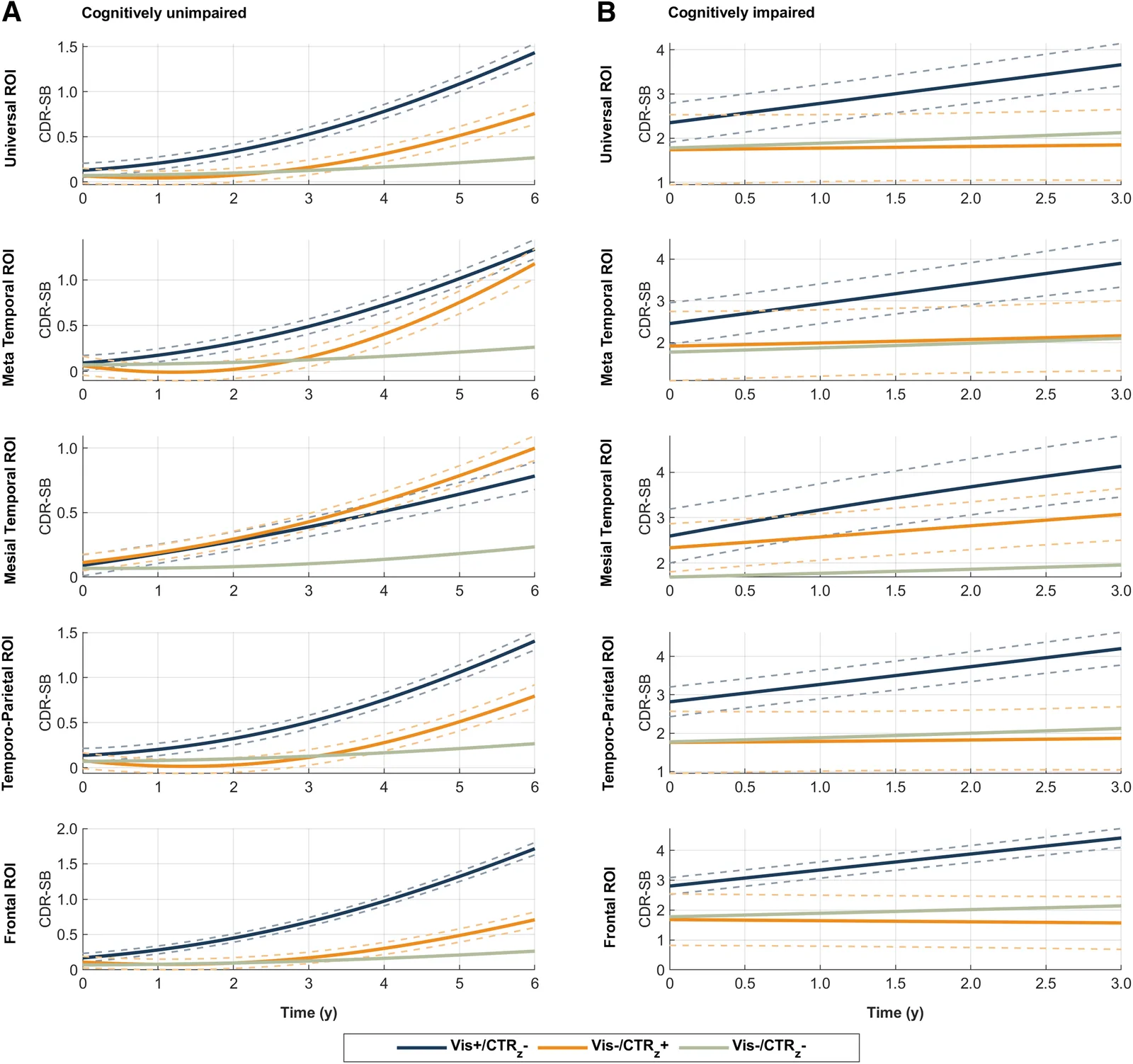
Trajectories on CDR-SB score for groups with discordant visual read/CenTauR_z_ tau PET status. Shown are average trajectories over time on CDR-SB score for groups with Vis+/CTR_z_− as well as groups with Vis−/CTR_z_+. Concordant group (Vis−/CTR_z_−) was included for reference purposes. Dashed lines represent 95% CIs.

## DISCUSSION

The present study investigated the concordance between 2 different definitions of tau PET positivity: the FDA/EMA-approved visual interpretation method for [^18^F]flortaucipir PET and CenTauR_z_-based quantification. Our findings indicate substantial, though not complete, agreement between the 2 methods in CI individuals and markedly lower agreement in CU individuals. Vis+ was more strongly associated with the presence of Aβ pathology and with significant clinical progression over time, even in CTR− cases. Together, these findings reveal inherent sensitivity differences between visual and quantification methods with regard to heterogeneous tau accumulation patterns, particularly in the earliest, presymptomatic disease stages.

The analysis of concordance between FDA/EMA-approved visual reads and CenTauR_z_-based quantification (Figs. 1 and 2) indicates a limited-to-moderate agreement between the 2 definitions of tau PET positivity, regardless of the ROI used for quantification. Notably, these results were not dependent on the prespecified CenTauR_z_ cut point of 2 and remained similar when using a Youden-based cut point to maximize agreement with visual reads, underscoring the inherent differences between the methods beyond simple cut point selection. Concordance was particularly low in CU individuals, with CenTauR_z_ quantification methods failing to capture 40%–80% of the visually positive cases. We noted that the low prevalence of visually positive scans in CU individuals (∼10%) may partly lower Cohen κ values despite relatively high overall agreement. Concordance was significantly higher among CI participants (κ = 0.57–0.82); notably, the Meta Temporal ROI showed slightly higher concordance than other ROIs, suggesting it may capture tau PET positivity more reliably; however, approximately 10%–40% of the Vis+ cases were not detected with CenTauR_z_ quantification. The Frontal ROI consistently showed lower concordance, likely expected given the limited contribution of frontal signal to the definition of Vis+ and its relatively minor involvement in early, heterogeneous tau deposition patterns. These findings suggest that visual read– and CenTauR_z_ quantification–based definitions of tau PET positivity cannot fully be used interchangeably.

The discordance between methods may primarily be attributed to the inability of CenTauR_z_ quantification to capture tau accumulation patterns that fall outside prespecified target ROIs but are easily detected by visual interpretation (Vis+/CTR_z_−; Fig. 4). Our findings also indicate that such noncanonical tau deposition patterns are not uncommon and can substantially affect the detection of early tau pathology, a limitation that likely extends to other ROI-based quantification approaches underlying current tau PET harmonization efforts. Importantly, Vis+/CTR_z_− cases showed high rates of Aβ positivity (75%–90%), supporting the biologic meaningfulness of this discordance and indicating the presence of AD-related tau pathology rather than off-target binding or visual misclassification. Collectively, these results highlight the higher sensitivity of visual reads for detecting relevant tau deposition patterns that deviate from canonical Braak-like spatial progression. Findings in the Mesial Temporal ROI should be interpreted cautiously, given the known partial-volume effects and tracer-specific limitations of [^18^F]flortaucipir in this region (12). A secondary, less common contributor to Vis+/CTR_z_− discordance may be off-target signal in the reference region, which can reduce SUVRs and lead to negative quantification despite visually detectable tau.

In contrast, Vis−/CTR_z_+ cases were significantly less frequent and showed weaker alignment with Aβ (Fig. 6), raising the possibility that a significant fraction of these cases represent noise or off-target binding rather than early tau pathology. However, because the FDA/EMA-approved visual interpretation method for [^18^F]flortaucipir does not consider anterior and mesial temporal regions, a subset of Vis−/CTR_z_+ individuals may indeed harbor early AD-related tau pathology in the mesial temporal lobe that would be missed and thus classified as Vis−.

The clinical implications of discordance differed across Vis+/CTR_z_− and Vis−/CTR_z_+ groups. Vis+/CTR_z_− individuals consistently demonstrated greater decline than those with Vis−/CTR_z_+, in both the CU and CI groups, reinforcing that visual reads capture prognostically relevant signal not detectable by CenTauR_z_. Vis−/CTR_z_+ individuals showed only modest or no differences compared with the Vis−/CTR_z_− reference group (Fig. 7), supporting the notion that the finding of CTR_z_+ in these cases may be largely driven by noise or off-target binding, with small negative effects likely reflecting statistical variability rather than true clinical improvement. Taken together, these findings indicate that, in cases of discordance, visual interpretation should be prioritized over fixed ROI–based quantification, particularly when assessing early tau pathology and its prognostic implications. Looking forward, hybrid or pattern-based quantification approaches that incorporate voxelwise, asymmetry-sensitive, or visually guided metrics could improve sensitivity and provide complementary information to enhance both research and clinical assessments of tau pathology.

This study had several limitations. First, we lacked neuropathologic confirmation and therefore cannot definitively determine which method most accurately reflects true tau pathology. Instead, we relied on indirect validation through relevant pathologic (Aβ status) and clinical (longitudinal cognitive decline) outcomes. Second, the cohort characteristics may limit generalizability, as our sample may differ from real-world populations with respect to racial composition, as well as the prevalence and stage of underlying pathology. Third, not all cohorts strictly adhered to the recommended 80–100-min postinjection acquisition window for [^18^F]flortaucipir PET (Supplemental Table 1), which may have introduced variability; however, this would affect both visual reads and quantitative measures, although not equally. Fourth, integrating multiple cohorts acquired across different scanners and sites may have added heterogeneity and noise, potentially challenging quantification approaches that rely on a single global cut point. Fifth, the visual read method used followed the FDA/EMA-approved standard; expert interpretations using alternative or more nuanced strategies may yield slightly different results. Finally, the CenTauR_z_ quantification pipeline is being updated to the CenTauR framework (22); while ROIs remain identical, new cut points and quantification methods may slightly alter the classification of some scans.

## CONCLUSION

Our results demonstrated that visual read and CenTauR_z_-based definitions of tau PET positivity are not interchangeable. Visual reads show closer alignment with Aβ status, identify clinically meaningful patterns of tau deposition that are missed by the commonly used fixed ROI/SUVR-based quantification methods (e.g., CenTauR_z_), and provide stronger prognostic value, particularly in cognitively unimpaired individuals. Our findings suggest that visual interpretation is more sensitive to early, regionally heterogeneous tau accumulation. Collectively, we highlight the need for quantitative approaches that move beyond fixed, predefined bilateral ROI masks.

## DISCLOSURE

Michel Grothe and Jesús Silva-Rodríguez are supported by research grants from the Institute of Health Carlos III–European Regional Development Fund (ISCIII-FEDER: PI24/00089) and the Reina Sofia Foundation (E.6.4 FRS-CUERPOS DE LEWY). Clifford Jack Jr. reports funding from National Institutes of Health (NIH) (grant R37 AG011378). Michael Schöll reports funding from the Knut and Alice Wallenberg Foundation (Wallenberg Centre for Molecular and Translational Medicine; KAW2014.0363 and KAW2023.0371), the Swedish Research Council (2021-06545 and 2023-06188), the European Union’s Horizon Europe research and innovation program under grant agreements 101132933 (AD-RIDDLE) and 101112145 (PROMINENT), NIH (R01 AG081394-01), Gates Ventures, National Research Foundation of Korea (RS-2023-00263612), Swedish governmental funding of clinical research, ALF agreement (ALFGBG-965326), Swedish Brain Foundation (FO2024-0372), Swedish Alzheimer Foundation (AF-1011738), JM Dahl Foundation, Familjen Rönström’s Foundation, Sahlgrenska Academy at the University of Gothenburg, Västra Götaland Region R&D (VGFOUREG-995510) and Innovation platforms, and Sahlgrenska Science Park. Alexis Moscoso serves as consultant to the Critical Path for Alzheimer Disease Consortium and CSO of ZRO Imaging. Valle Camacho has received honoraria for presentations or educational material from GE HealthCare, Eli Lilly and Co., Life Molecular Imaging, and Novartis. Jesús Silva-Rodríguez is a consultant, stakeholder, and board member of Qubiotech Health Intelligence S. L. Andres Perissinotti has received honoraria for presentations or educational material from GE HealthCare. Nicolai Franzmeier received consulting, speaker or travel honoraria from MSD, Life Molecular Imaging, GE HealthCare, Eisai, Eli Lilly and Co., MSD, Lantheus, and Biogen and research support from Avid Radiopharmaceuticals. Giovanni Frisoni has received competitive research funds from H2020, Innovative Medicines Initiative (IMI), IMI2, Innovative Health Initiative (IHI), Swiss National Science Foundation, VELUX Foundation, and Synapsis Foundation and unrestricted grants and support for event organization from Hoffman-La Roche, OM Pharma, Eisai, Vifor Pharma, OM Pharma, Biogen Pharmaceuticals, Novo Nordisk, GE HealthCare, Eli Lilly and Co., and Life Molecular Imaging. Valentina Garibotto received research support and speaker fees through her institution from GE HealthCare, Siemens Healthineers, Eli Lilly and Co., Novartis, and Roche. Ronald Petersen discloses relationships with Roche, Genentech, Eli Lilly and Co., Eisai, Novartis, and Novo Nordisk. Clifford Jack Jr. is employed by Mayo Clinic and receives research support from NIH, GHR Foundation, and Alexander Family Alzheimer Disease Research Professorship of the Mayo Clinic. PV reported grants from the NIH. Michael Schöll has served/serves as advisor to Arvakor, Eli Lilly and Co., Gates Ventures, Novo Nordisk, Ribocure and Roche, received honoraria for presentations or educational material from Bioarctic, Novo Nordisk, Eli Lilly and Co., Roche and Triolabs, receives research support (to the institution) from Beckman Coulter, Bioarctic, Gates Ventures, Novo Nordisk and Roche, and is cofounder and shareholder of Centile Bioscience. Data used in the preparation of this article was obtained from multiple international cohorts, including the Mayo Clinic Study of Aging, Alzheimer’s Disease Neuroimaging Initiative, Anti-Amyloid Treatment in Asymptomatic Alzheimer’s Study, OASIS-3 trial, the Avid Radiopharmaceuticals ^18^F-AV-1451-A05 study, National Alzheimer’s Coordinating Center’s Standardized Centralized Alzheimer’s and Related Dementias Neuroimaging initiative, Mayo Clinic Alzheimer’s Disease Research Center, Geneva Memory Center, Harvard Aging Brain Study, and Samsung Medical Center. Detailed cohort-specific acknowledgments and funding information are provided in the supplemental materials. No other potential conflict of interest relevant to this article was reported.

## Notes

KEY POINTS **QUESTION:** Does the CenTauR quantification method for [^18^F]flortaucipir PET imaging align with the FDA/EMA-approved visual interpretation method? **PERTINENT FINDINGS:** In a cohort study of 3991 participants, concordance between visual interpretation and CenTauR_z_ quantification was limited, especially in CU individuals. Visual reads correlated better with Aβ positivity, faster clinical progression, and provided stronger prognostic value than did CenTauR_z_ quantification. **IMPLICATIONS FOR PATIENT CARE:** Visual tau PET interpretation may offer higher sensitivity to early tau pathology, underscoring the need for improved quantification methods for early AD detection and monitoring.

## References

[1] Fleisher AS Pontecorvo MJ Devous MD Sr.; A16 Study Investigators. Positron emission tomography imaging with [^18^F]flortaucipir and postmortem assessment of Alzheimer disease neuropathologic changes. JAMA. Neurol. 2020;77:829–839. 10.1001/jamaneurol.2020.0528 PMC7186920 32338734

[2] Soleimani-Meigooni DN Iaccarino L La Joie R. ^18^F-flortaucipir PET to autopsy comparisons in Alzheimer’s disease and other neurodegenerative diseases. Brain. 2020;143:3477–3494. 10.1093/brain/awaa276 PMC7719031 33141172

[3] Nelson PT Jicha GA Schmitt FA. Clinicopathologic correlations in a large Alzheimer disease center autopsy cohort: neuritic plaques and neurofibrillary tangles “do count” when staging disease severity. J Neuropathol Exp Neurol. 2007;66:1136–1146. 10.1097/nen.0b013e31815c5efb PMC3034246 18090922

[4] Tauvid prescribing information. Food and Drug Administration website. https://www.accessdata.fda.gov/drugsatfda_docs/label/2020/212123s000lbl.pdf. Accessed April 13, 2025.

[5] Tauvid product information. European Medicines Agency website. https://www.ema.europa.eu/en/medicines/human/EPAR/tauvid. Accessed April 13, 2025.

[6] Lu M Pontecorvo MJ Devous MD Sr.; AVID Collaborators. Aggregated tau measured by visual interpretation of flortaucipir positron emission tomography and the associated risk of clinical progression of mild cognitive impairment and Alzheimer disease: results from 2 phase III clinical trials. JAMA Neurol. 2021;78:445–453. 10.1001/jamaneurol.2020.5505 PMC7885097 33587110

[7] Chen CD Ponisio MR Lang JA. Comparing tau PET visual interpretation with tau PET quantification, cerebrospinal fluid biomarkers, and longitudinal clinical assessment. J Alzheimers Dis. 2023;93:765–777. 10.3233/JAD-230032 PMC10200228 37092225

[8] Soleimani-Meigooni DN Smith R Provost K. Head-to-head comparison of tau and amyloid positron emission tomography visual reads for differential diagnosis of neurodegenerative disorders: an international, multicenter study. Ann Neurol. 2024;96:476–487. 10.1002/ana.27008 PMC11324380 38888212

[9] Groot C Smith R Collij LE. Tau positron emission tomography for predicting dementia in individuals with mild cognitive impairment. JAMA Neurol. 2024;81:845–856. 10.1001/jamaneurol.2024.1612 PMC11165418 38857029

[10] Gogola A Minhas DS Villemagne VL. Direct comparison of the tau PET tracers ^18^F-flortaucipir and ^18^F-MK-6240 in human subjects. J Nucl Med. 2022;63:108–116. 10.2967/jnumed.120.254961 PMC8717175 33863821

[11] Mummery CJ Li-Hsian CC Lasagna-Reeves CA. Tau in Alzheimer’s disease: shaping the future patient journey. J Prev Alzheimers Dis. 2026;13:100447. 10.1016/j.tjpad.2025.100447 PMC12869042 41478818

[12] Villemagne VL Leuzy A Bohorquez SS. CenTauR: toward a universal scale and masks for standardizing tau imaging studies. Alzheimers Dement (Amst). 2023;15:e12454. 10.1002/dad2.12454 PMC10326476 37424964

[13] Leuzy A Raket LL Villemagne VL. Harmonizing tau positron emission tomography in Alzheimer’s disease: the CenTauR scale and the joint propagation model. Alzheimers Dement. 2024;20:5833–5848. 10.1002/alz.13908 PMC11497758 39041435

[14] Klunk WE Koeppe RA Price JC. The Centiloid Project: standardizing quantitative amyloid plaque estimation by PET. Alzheimers Dement. 2015;11:1–15e4. 10.1016/j.jalz.2014.07.003 PMC4300247 25443857

[15] Jack CR Jr Wiste HJ Weigand SD. Defining imaging biomarker cut points for brain aging and Alzheimer’s disease. Alzheimers Dement. 2017;13:205–216. 10.1016/j.jalz.2016.08.005 PMC5344738 27697430

[16] Weigand AJ Maass A Eglit GL Bondi MW. What’s the cut point?: a systematic investigation of tau PET thresholding methods. Alzheimers Res Ther. 2022;14:49. 10.1186/s13195-022-00986-w PMC8985353 35382866

[17] Albert MS DeKosky ST Dickson D. The diagnosis of mild cognitive impairment due to Alzheimer’s disease: recommendations from the National Institute on Aging–Alzheimer’s Association workgroups on diagnostic guidelines for Alzheimer’s disease. Alzheimers Dement. 2011;7:270–279. 10.1016/j.jalz.2011.03.008 PMC3312027 21514249

[18] McKhann GM Knopman DS Chertkow H. The diagnosis of dementia due to Alzheimer’s disease: recommendations from the National Institute on Aging–Alzheimer’s Association workgroups on diagnostic guidelines for Alzheimer’s disease. Alzheimers Dement. 2011;7:263–269. 10.1016/j.jalz.2011.03.005 PMC3312024 21514250

[19] La Joie R Ayakta N Seeley WW. Multisite study of the relationships between antemortem [^11^C]PIB-PET Centiloid values and postmortem measures of Alzheimer’s disease neuropathology. Alzheimers Dement. 2019;15:205–216. 10.1016/j.jalz.2018.09.001 PMC6368897 30347188

[20] Moscoso A Heeman F Raghavan S.; PREVENT-AD Research Group, the Harvard Aging Brain Study, the LEADS Consortium, and the Alzheimer’s Disease Neuroimaging Initiative. Frequency and clinical outcomes associated with tau positron emission tomography positivity. JAMA. 2025;334:229–242. 10.1001/jama.2025.7817 PMC12848857 40522652

[21] O’Bryant SE Waring SC Cullum CM. Staging dementia using Clinical Dementia Rating Scale Sum of Boxes scores: a Texas Alzheimer’s Research Consortium study. Arch Neurol. 2008;65:1091–1095. 10.1001/archneur.65.8.1091 PMC3409562 18695059

[22] Moscoso A Leuzy A Raket LL. External validation of joint propagation model‐based tau PET CenTauR units. Alzheimers Dement. 2026;21(suppl 2):e106362.

